# Epigenetic aging signatures in mice livers are slowed by dwarfism, calorie restriction and rapamycin treatment

**DOI:** 10.1186/s13059-017-1186-2

**Published:** 2017-03-28

**Authors:** Tina Wang, Brian Tsui, Jason F. Kreisberg, Neil A. Robertson, Andrew M. Gross, Michael Ku Yu, Hannah Carter, Holly M. Brown-Borg, Peter D. Adams, Trey Ideker

**Affiliations:** 1 Department of Medicine, University of California San Diego, La Jolla, CA 92093 USA; 2 Beatson Institute for Cancer Research and University of Glasgow, Glasgow, UK; 3 Department of Biomedical Sciences, School of Medicine and Health Sciences, University of North Dakota, Grand Forks, ND 58202 USA; 4 Sanford Burnham Prebys Medical Discovery Institute, La Jolla, CA 92037 USA; 5 Bioinformatics and Systems Biology Program, University of California San Diego, La Jolla, CA 92093 USA

**Keywords:** DNA methylation, Epigenomics, Aging, Epigenetic aging

## Abstract

**Background:**

Global but predictable changes impact the DNA methylome as we age, acting as a type of molecular clock. This clock can be hastened by conditions that decrease lifespan, raising the question of whether it can also be slowed, for example, by conditions that increase lifespan. Mice are particularly appealing organisms for studies of mammalian aging; however, epigenetic clocks have thus far been formulated only in humans.

**Results:**

We first examined whether mice and humans experience similar patterns of change in the methylome with age. We found moderate conservation of CpG sites for which methylation is altered with age, with both species showing an increase in methylome disorder during aging. Based on this analysis, we formulated an epigenetic-aging model in mice using the liver methylomes of 107 mice from 0.2 to 26.0 months old. To examine whether epigenetic aging signatures are slowed by longevity-promoting interventions, we analyzed 28 additional methylomes from mice subjected to lifespan-extending conditions, including Prop1^df/df^ dwarfism, calorie restriction or dietary rapamycin. We found that mice treated with these lifespan-extending interventions were significantly younger in epigenetic age than their untreated, wild-type age-matched controls.

**Conclusions:**

This study shows that lifespan-extending conditions can slow molecular changes associated with an epigenetic clock in mice livers.

**Electronic supplementary material:**

The online version of this article (doi:10.1186/s13059-017-1186-2) contains supplementary material, which is available to authorized users.

## Background

In humans, numerous CpG sites have DNA methylation states that correlate with age. These associations have been used to formulate models, called epigenetic clocks, that make quantitative predictions of age based on selected sets of CpG sites [1–3]. These models are derived from the methylation profile of many individuals measured using oligonucleotide arrays, such as the Illumina 450 K platform, which determines the methylation value at >450,000 CpG sites genome-wide. Although the age predictions of these molecular models are generally very accurate across the human population, for particular individuals the prediction can be markedly different from the actual chronological age. For example, an advanced molecular age relative to chronological age has been associated with a number of diseases, such as obesity, viral infection and Down syndrome [4–6]. Furthermore, a recent retrospective analysis of longitudinal cohort studies showed that a molecular age advancement of 5 years corresponded to a 21% increased risk of mortality overall [7]. Thus, predictions of “epigenetic age” may be an indication of an individual’s biological state of aging.

Beyond these examples of advanced epigenetic aging, a complementary but unanswered question is whether epigenetic clocks can also be slowed. Epigenetic aging studies in humans have not thus far been well suited to address questions of slowed aging, given the lack of well-documented interventions that enhance health or lifespan and the difficulty of controlling for confounding factors. However, rodents are particularly appealing experimental organisms in studies of mammalian aging, because they are genetically tractable and can be subjected to potential lifespan-extending interventions. The earliest described such intervention, calorie restriction, was shown to extend rodent lifespan by as much as 2-fold [8]. These findings have since been replicated in numerous mouse strains [9]. Another well-studied lifespan-extending condition is a single-point mutation in the Prop1 gene that results in dwarfism and lifespan extension up to 1.5-fold [10]. These effects are likely due to reduced somatotropic signaling [11]. A more recently described treatment, dietary rapamycin, has been reported to increase the lifespan of genetically heterogeneous mice by 1.2-fold [12].

Despite these known lifespan-extending interventions, an epigenetic clock has not yet been formulated for mice. Nonetheless, mouse methylation signatures can now be analyzed genome-wide using either reduced representation bisulfite sequencing (RRBS) or whole genome bisulfite sequencing (WGBS) [13]. Using such data, previous studies have suggested that mice might experience patterns of epigenetic aging similar to those documented in humans [14–16]. For instance, CpG methylation sites distinguish young versus old mouse hematopoietic stem cells [17], and CpG methylations altered in murine acute myeloid leukemia are also found to change with age [18]. These findings suggest that an epigenetic measure of age is plausible for mice.

Here, we ask if conditions that extend mouse lifespan – Prop1^df/df^ dwarfism, calorie restriction and dietary rapamycin – also affect a mouse epigenetic clock. While such a link seems plausible, an alternative possibility is that these lifespan-extending conditions might operate independently of the changes that underlie an epigenetic clock, which would then proceed at a normal rate despite intervention. To distinguish between these possibilities, we first assess whether there are similarities between mouse and human epigenetic aging. We then formulate epigenetic readouts of age to score the effect of lifespan-extending interventions.

## Results

### Age-related methylation changes share common behavior in mouse and human

First, we assessed the similarities of age-related methylome changes between mice and humans. For this purpose, we obtained publicly available mouse methylation data from Reizel et al. [19] consisting of RRBS from livers of 102 male or female C57BL/6 mice ranging in age from 0.2 to 7.1 months. These data were filtered to identify sites that were reliably measured with sufficient sequencing depth in most mice (see “Methods”), yielding 36,094 CpG sites total, of which 27,61﻿2 CpG sites were conserved in human﻿s.

**Fig. 1:**
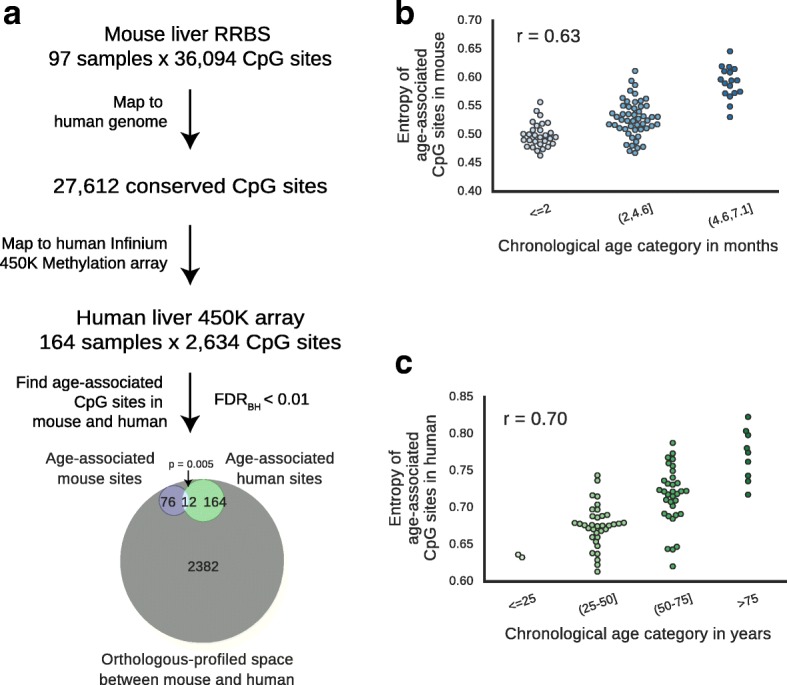
Comparison of methylation aging in mice and human livers. **a** Mapping from mouse CpG sites profiled by reduced representation bisulfite sequencing (*RRBS*) to orthologous CpG sites profiled by Illumina 450 K human methylation array. Detailed procedures can be found in “Methods.” The Venn diagram describes the age-associated sites in the orthologous–profiled space. **b**, **c** Entropy across all age-associated sites in mouse (b) and in humans (c) is plotted over age. Pearson’s correlation (r) is displayed (mouse *p* < 10^−11^, human *p* < 10^−11^). *FDR* false discovery rate

 Next, we obtained publicly available methylation data from 164 human livers that were generated using 450 K Illumina methylation arrays [4, 20]. We identified 2634 CpG sites that were assayed in the human Illumina arrays that were orthologous to those from the set of filtered sites from mouse RRBS (Fig. 1a). From this orthologous-profiled space, we identified 88 age-associated sites in mice (for which the methylation status had a significant association with age) and 176 age-associated sites in humans (Fig. 1a, likelihood ratio test at 1% false discovery rate (FDR), see “Methods”). Among these, we saw slight but significant overlap between sites that were age-associated in mice versus sites that were age-associated in humans (Fig. 1a) (*p* < 0.01 by hypergeometric test). Notably, the age-associated sites in both species showed similar under/over-enrichments in various genomic annotations, including regions marked by histones (H3K27me3, bivalent and H3K9ac), although different genomic regions were associated with statistical significance in mice and humans, with only H3K27ac regions significantly under-enriched in both species (Additional file 1: Figure S1)*.* Thus, age-associated CpG sites in the orthologous-profiled space appear to be slightly conserved with respect to various genomic regions affected.

Previous methylation studies of whole blood in humans have documented increasing entropy with age [1, 5]. Increasing entropy indicates that, during aging, the state of each CpG becomes less uniform across the cell population [1]. We asked if this trend of increasing disorder of age-associated CpG sites in the methylome also exists in mice and human livers, regardless of whether a particular site was sampled in both species. We saw that, in both mice and humans, the age-associated regions of the methylome tended toward higher disorder (Fig. 1b, c). This finding suggests that a trend toward disorder over time is a conserved property of aging in mammals.

### Development of an epigenetic clock in mice

Motivated by the shared patterns affecting the aging epigenome in mice and humans, we next formulated an epigenetic clock for mice. Toward this goal, we created a consolidated mouse liver methylome dataset combining two previous studies [19, 21] with data newly generated in this study (Additional file 2: Datasets used summary). This consolidated dataset consisted of 107 liver methylomes of mice aged 0.2 to 26.0 months old (Additional file 1: Figure S2A), covering 7628 CpG sites that were detected in nearly all samples (“Methods”). Normalization with ComBat [22, 23] was performed to estimate and remove effects resulting from the different sequencing technologies (RRBS and WGBS) and mouse strains (Ames, C57BL/6 and UM-HET3) in this integrated dataset (“Methods”). To train a predictive model of mouse age that can be used as an epigenetic clock, we applied ElasticNet [24], a statistical regression framework used previously to formulate epigenetic clocks in humans [1, 2]. This training process selected a subset of 148 CpG sites for an epigenetic clock in mice livers (Additional file 3). These sites were predominantly located in intronic and intergenic regions and, in particular, were significantly under-represented in promoters and over-represented in enhancers (Additional file 1: Figure S2B, *p* < 0.01 and *p* < 10^-5^ by Fisher’s exact test, respectively)*.*

**Fig. 2:**
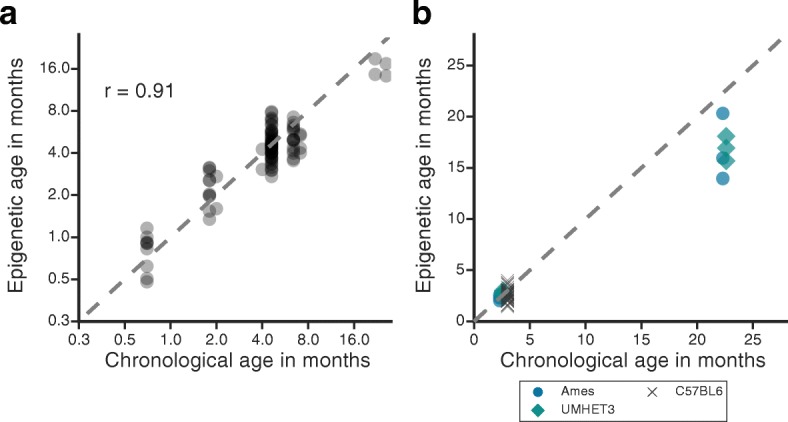
Validation of an epigenetic-aging model in mice livers. **a** Four-fold cross validation of the age predictions (y-axis, “Epigenetic age”) versus chronological age (x-axis) in log_2_ scale. Each *dot* represents a prediction made for a single mouse. Overall, each fold has a high Pearson’s correlation (r) between epigenetic and chronological age, and the average among all folds is depicted. **b** Epigenetic ages versus chronological ages for 50 wild-type mice. Different *symbols/colors* are used to indicate the mouse genetic background. The *dashed line* represents the diagonal in both plots

We pursued two different strategies to assess predictive performance. First, we performed 4-fold cross validation, in which the 107 mice used for training were arbitrarily divided into four sets of comparable sizes. Each of these sets was withheld, in turn, from model training and instead used to test the performance of the trained model. In this cross-validation scenario, we found that the ages of the test sets were accurately predicted with a correlation ranging from 83% to 92% (average r = 0.91; Fig. 2a). Second, we tested the performance of the model when predicting age from the liver methylomes of 50 mice that had not been used for model training or cross validation (spanning three mouse strains and two ages, 2 and 22 months; Additional file 2: Datasets used summary). Predicted epigenetic ages were well correlated with chronological ages (Fig. 2b) and did not show any strain-specific effects: 2-month-old Ames wild-type, UM-HET3 and C57BL/6 mice had roughly the same epigenetic age; the same was true for 22-month-old Ames wild-type and untreated UM-HET3 mice, with an average prediction error of 4.2 months (Additional file 1: Figure S2C, Additional file 4: Wild type mice predictions & Wild type stats).

### Lifespan extension slows epigenetic aging

**Fig. 3:**
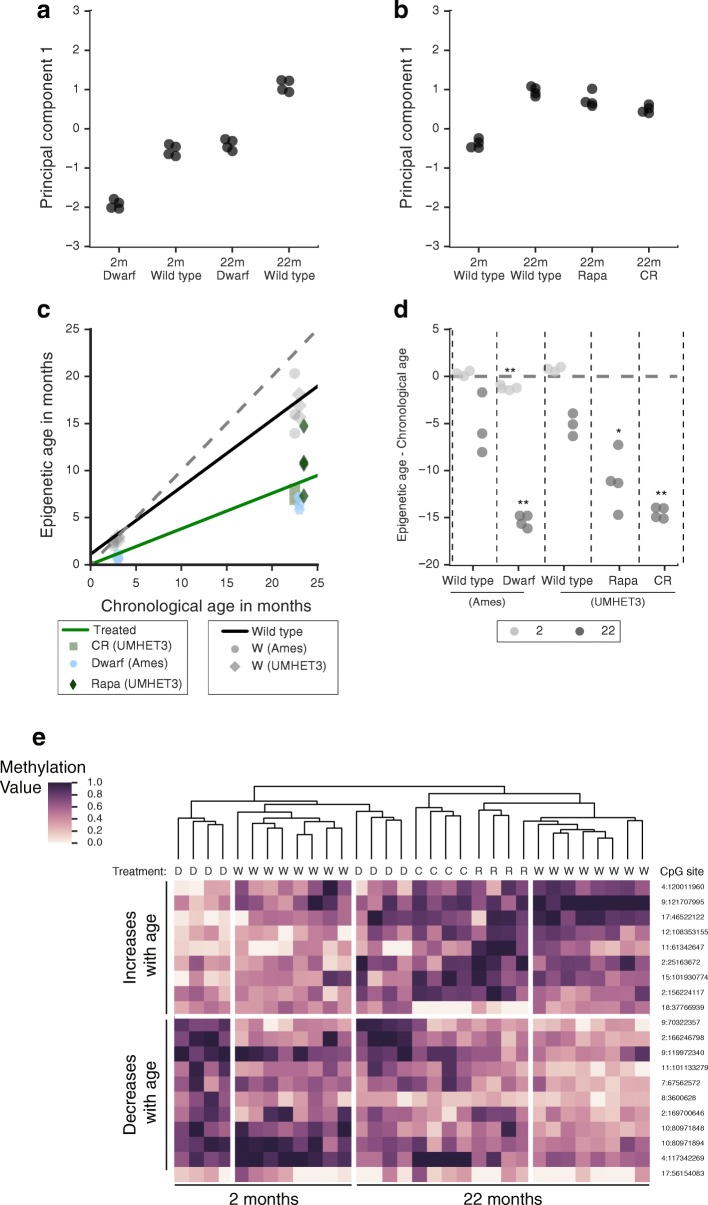
Effects of lifespan extension on a mouse epigenetic clock. **a**, **b** The 148 CpG sites used in the mouse epigenetic-aging model (used for a mouse epigenetic clock) were subjected to principal component analysis. Principal component 1 is plotted for wild-type mice according to age and lifespan extension status, for wild-type Ames or dwarf mice (a) or wild-type UM-HET3, rapamycin-treated or calorie-restricted mice (b). **c** The mouse epigenetic-aging model applied to long-lived mice, with *colors* and *shapes* representing the different lifespan-enhancing conditions. The *gray markers* are the wild-type mice (identical to Fig. 2b), and the *black line* represents the linear fit of the epigenetic age versus chronological age of the wild-type mice. The *green line* represents the linear fit of the epigenetic age versus chronological age for long-lived mice. The *gray dashed line* represents the diagonal. **d** The residual (epigenetic age minus chronological age) is plotted for all mice according to their strain and treatment, and *colors* represent 2 or 22 months of age. *p*-values were calculated by comparing ages of long-lived mice to age-matched controls of the same genetic background using a t-test. *p < 0.05; **p < 0.01. **e** Hierarchical clustering of the top 20 most variable sites used by this epigenetic clock using average linkage with Euclidean distance. Treatment is depicted under the dendrogram, CpG sites are to the right of the heatmap (chromosome:start, 0-based) and rows are blocked according to clusters of sites that increase or decrease methylation with age. *m* Months, *R* Rapamycin treatment, *C*, *CR* Calorie restriction, *D* Ames Dwarf, *W* wild-type Ames or untreated, wild-type UM-HET3

We then assessed the behavior of these 148 CpG sites in the WGBS data generated from mice subjected to various lifespan-extending conditions. This analysis included methylomes from Prop1^df/df^ dwarf mice at 2- or 22-months-old [10], with four in each group; four calorie-restricted mice at 22-months-old; four rapamycin-treated mice at 22-months-old [12], and the control mice of the same genetic background described above. First, we used principal component analysis (PCA) using these CpG sites (Additional file 1: Figure S3A). The first principal component of these features (PC1) correlated strongly with age, and PC1 values of mice subjected to lifespan-extending treatments were always lower than PC1 values of age-matched controls (Fig. 3a, b; Additional file 1: Figure S3A; Additional file 5). Next, we applied the epigenetic-aging model to these mice (“Methods”; Additional file 2: Datasets used summary; Additional file 4: Long-lived mice predictions). We found that the predicted epigenetic ages of these long-lived mice were significantly less than those of age-matched control mice (Fig. 3c). Reinforcing this observation, such differences were also detected by an ANOVA statistical analysis between the lifespan-extending conditions versus control mice aged to 22 months (*p* < 10^−4^; “Methods”; Additional file 4: Treatment vs wild type stats). In particular, an average reduction of 10.1 months was seen when comparing the epigenetic ages of 22-month-old dwarf mice to 22-month-old wild types (*p* < 0.01 by t-test, Fig. 3d). Similar reductions in epigenetic ages were observed in calorie-restricted mice versus their age-matched controls, corresponding to a 9.4-month decrease on average (*p* < 10^−4^, Fig. 3d). Rapamycin treatment had a smaller, but significant effect on epigenetic ages, corresponding to a 6.0-month decrease on average in rapamycin-treated mice compared to age-matched controls (*p* < 0.05, Fig. 3d). Finally, 2-month-old dwarf mice also had reduced epigenetic ages compared to 2-month-old wild-type mice, by 1.5 months on average (*p* < 10^−3^, Fig. 3d). These results are consistent with the smaller magnitudes of age-associated PC1 of long-lived mice, relative to their age-matched controls.

We then assessed the change in methylation with age of the 148 CpG sites used to formulate this epigenetic clock. Among these CpG sites, we found that 76 gained methylation with age and 72 lost methylation with age. These sites clustered the mice according to age and treatment rather than by genetic background (Fig. 3e; Additional file 1: Figure S3B). Among CpG sites whose methylation decreased with age, we saw that long-lived mice generally had higher methylation values than their age-matched controls, which may have contributed toward the observed decreases in epigenetic age (Fig. 3e). Thus, whether examined individually (Fig. 3e) or summarized along a single dimension (Fig. 3a, b), changes in methylation due to aging are generally less extreme in mice exposed to pro-longevity conditions, leading to younger epigenetic ages (Fig. 3c, d).

## Discussion

Previous studies in humans have shown that epigenetic clocks can be accelerated by conditions associated with decreased lifespan [4–6]. However, it was unclear if these epigenetic clocks could be slowed by conditions that increase lifespan. Here, we have found that lifespan-extending interventions can indeed slow an epigenetic clock in mice livers. Previous studies of these longevity-promoting interventions have shown that these interventions not only extend lifespan [9, 10, 25], but also improve tissue and physical functioning with age [26–29]. Interestingly, rapamycin had a smaller effect than the other treatments considered here, possibly due to metabolic differences, such as increased insulin resistance under rapamycin treatment [30]. Nonetheless, our findings suggest that epigenetic clocks, measured from DNA methylation, can be slowed by lifespan-extending conditions.

Notably, we found that dwarf mice had a decreased epigenetic age at our earliest time point, when just 2 months old (Fig. 3c,d). This finding suggests that age-related changes in the methylome occur during both development and aging [31–34]. Prominent changes in the DNA methylome have been observed during development in mice and continue gradu﻿ally throughout adulthood [33]. In humans, epigenetic clocks are accurate in both adolescents and adults [2]. Thus, the decrease in epigenetic age of young dwarf mice is consistent with their apparent developmental delay [11].

Comparing this epigenetic clock to those in humans, we observed a prediction error of 4.2 months (~16% relative to the average mouse lifespan), whereas that for human clocks was 3.7 years [1, 2] (~5% relative to the average human lifespan). This difference in accuracy is likely due to two major technical differences. The first is that there are fewer samples available in mice than there are for humans. Second, the methylation profiles from mice represent a random sampling of genomic regions (RRBS and WGBS), whereas those from humans derive from microarrays, in which a consistent set of sites is reproducibly measured. However, when comparing age-related methylation changes between mice and humans, we found that the age-associated methylome exhibits increased disorder in both species (Fig. 1b, c). These results suggest that, regardless of the specific regions impacted, the increased disorder of the age-associated methylome is a common feature of mammalian aging. This increased disorder of the age-associated methylome may contribute to our ability to formulate epigenetic clocks in both species [1–3, 35].

Finally, since this mouse clock was developed using liver methylomes, in future studies it will be very interesting to examine whether these clocks are similar across various tissues. Intriguingly, previous studies in humans have found that obesity is specifically associated with epigenetic age advancement in the liver but not in other tissues such as blood [4]. Furthermore, rapamycin treatment has been shown to accelerate cataract formation in eyes and increase testicular degeneration, but delays age-related phenotypes in other tissues [29]. A key question will be whether these same tissue-specific effects are reflected in epigenetic aging rates, in which some tissues may reflect slowed aging while others reflect accelerated aging.

## Conclusions

We have formulated an epigenetic-aging model in mice and used it to find evidence that lifespan-extending conditions slow an epigenetic clock in mice livers. To further understand whether lifespan-extending conditions promote more youthful epigenetic signatures globally, it will be of interest to study different tissues, as well as profile mice exposed to other lifespan-extending conditions, such as methionine restriction or other mutations in somatotropic signaling pathways [26]. Ultimately, such studies will help elucidate the relationship between the slowed epigenetic clock and healthy aging.

## Methods

### Long-lived mice

To study the effects of dwarfism, we studied 2- or 22-month-old male Ames Prop1^df/df^ dwarf and wild-type mice livers [10], with four mice in each group. Mice were maintained under controlled conditions at the University of North Dakota (﻿﻿Grand Forks, N﻿﻿D, USA﻿)﻿ with access to food ad libitum. To study the effects of calorie restriction and rapamycin treatment, we used female UM-HET3 mice livers aged to 22 months, where mice were subjected to calorie restriction (60% of food consumption relative to age-matched controls, gradually reduced over 2 weeks), subjected to 42 mg/kg dietary rapamycin treatment from 4 to 22 months, or left untreated, with four mice in each group. We also obtained livers from female untreated UM-HET3 mice aged to 2 months [12]. UM-HET3 mice were maintained at the University of Michigan (Ann Arbor, MI, USA). The weights of these mice are described in Additional file 6.

### WGBS library preparation

DNA was isolated from mice livers using the DNeasy blood and tissue kit (Qiagen, Germantown, MD, USA). WGBS was carried out by the Beijing Genomics Institute (Shenzhen, China) following standard protocols [36]. Briefly, DNA was fragmented using sonication to an average fragment size of 100–300 bp, end-repaired, and ligated to methylated-sequencing adapters to generate sequencing libraries. Bisulfite conversion was performed on these sequencing libraries using the ZYMO EZ DNA Methylation-Gold kit ﻿(Irvine, CA, US﻿A﻿) and sequenced using 90 bp paired-end sequencing on an Illumina HiSeq-4000 (San Diego, ﻿CA, USA). Ames mice were sequenced to an expected 15× coverage; UM-HET3 mice were sequenced to an expected  5× coverage.

### Data processing

For the WGBS study in long-lived mice, sequencing reads were trimmed using Trim Galore [37] and aligned to a bisulfite-converted mouse genome (mm9) obtained from UCSC [38] using bowtie [39]. Methylation states were called using bismark v0.10.0 [40]. The resulting sites were then converted to mm10 coordinates using liftOver [38] with default parameters.

In addition to the above data, public bisulfite sequencing data were downloaded from GEO [41] or the Sequence Read Archive (SRA) (accession numbers: [GEO: GSE60012] [19], [GEO: GSE52266] [42], [GEO: GSE67507] [43] and [SRA344045] [21]). Sequencing reads were trimmed using Trim Galore [37] with default parameters, aligned to bisulfite-converted Ensembl mmGRC38 version 84 [44] using bowtie2 [45] with parameters –N 1, and the methylation states were determined using Bismark v0.14.3 [40]. When multiple sequencing runs were associated with a single sample, the methylation states for each CpG were collapsed by summing the reads.

Human 450 K liver data were downloaded from GEO (accession numbers: [GEO: GSE61258] and [GEO: GSE48325]), corresponding to Horvath et al. [4] and Ahrens et al. [20] datasets. The data were processed in R using Minfi [46]. Missing data were imputed using impute package in R [47]. The data were then beta-mixture quantile normalized [48] using a gold reference distribution following the procedure provided by Horvath [2]. The gold reference distribution was set to the mean probe values from [GEO: GSE61258].

### Evolutionary trends

To compare mice with humans, we wanted to maximize the number of mouse CpG markers that we could compare reliably across species. For this reason, we limited our analysis to RRBS datasets obtained from GEO. Specifically, we filtered Reizel et al. [19] with Cannon et al*.* [42] and Orozco et al*.* [43] to identify reproducible CpG sites. Sites were filtered according to the following criteria: ≥5 reads, <20% missing data across mice from all three studies, and distinct mapping onto chromosomes 1–19. We then removed individual mouse samples missing >40% of these sites. These filtering steps resulted in 97 samples profiled across 36,094 sites in Reizel et al*.* [19]. Missing data were imputed using the mean methylation value for that site.

To define a commonly–profiled set of orthologous CpG sites, we mapped the 36,094 sites profiled in mm10 to hg19 coordinates using liftOver [38], with -minMatch = 0.1. The resulting coordinates were intersected with the Illumina 450 K probes, as defined by their locations from the Illumina manifest (bedtools intersectbed [49]). Any mouse sites that mapped to the same human site were combined by taking the average value of these sites.

Annotation tracks were downloaded from Encode for human hepatocytes from UCSC [50]. The following data tracks were downloaded: DNASE-seq, H3K36me3, H3K4me1, H3K27ac, H3K9ac, H3K4me3 and H3K27me3. Enhancer regions were defined as the intersected regions between H3K27ac and H3K4me1. Bivalent regions were defined as the intersected regions between H3K4me3 and H3K27me3. Repeat elements were downloaded from UCSC for hg19 [51]. CpG sites were mapped to each feature by intersecting the site coordinates with each annotation using bedtools intersectbed. Annotations for transcription start site (TSS), 5′ untranslated region (UTR), body, exons, shelf, island and shore were defined by the Illumina 450 K manifest. Promoters were defined as CpG sites with TSS annotations. Similarly for mice, annotation tracks were downloaded from UCSC for the same marks from adult male mice liver. Gene features for mice were also downloaded from UCSC for mm10 or mm9 [51]. Coordinates for mm9 were translated to mm10 using liftOver (default parameters) and assigned to sites using bedtools intersectbed. Promoters in mice were defined as 2 kb upstream of protein-coding genes. We only considered annotations that fell within the orthologous-profiled set of CpGs. These annotations were used as genomic regions.

Odds ratios (ORs) were calculated by counting orthologous CpG sites annotated to different genomic regions and assessing whether they were age-associated or not age-associated. This formed a 2-by-2 contingency table for each genomic region, so we could assess whether age-associated sites were under-represented or over-represented in that particular genomic region. This process was repeated for each genomic region separately in both human and mouse. When there were overlapping genomic region annotations for sites, sites were counted only for the genomic region considered so that sites were not counted twice. Over-represented genomic regions were those with an OR > 1 and under-represented genomic regions were those with an OR < 1. *p*-values were calculated using Fisher’s exact test.

To identify age-associated sites, we built a multivariate linear model regressing each methylation site against treatment, gender and age in mice, or against body mass index, gender and age in humans. Then, we conducted a drop-one F-test to determine if age had a significant association with that site. For comparisons in the orthologous-profiled space between mice and humans, we conducted the drop-one F-test using Reizel et al*.* [19] for mice or all human samples, and we selected sites that had an age-association at a Benjamini-Hochberg 1% FDR. To calculate the significance of the overlap, we used a hypergeometric test.

To identify all age-associated sites, regardless of conservation, we conducted the same drop-one F-test, first using the 97 mice of Reizel et al*.* [19] for all 36,094 CpG sites, then selecting CpG sites that passed a Benjamini-Hochberg 1% FDR. We repeated this analysis using the 2.1-month-old mice from Cannon et al*.* [42] and 3.7-month-old mice from Orozco et al*.* [43], using the CpG sites identified in Reizel et al*.* [19], and selected sites that continued to have an age-association at a Benjamini-Hochberg 1% FDR. Using these criteria, we found 393 age-associated sites in mice. These sites were used to calculate entropy for Reizel et al. [19] (Fig. 1b). We identified age-associated CpG sites in humans similarly, using all 485,512 CpG sites on the 450 K Illumina chip, first in [GEO: GSE61258] [4] (79 samples), identifying CpG sites with an age-association at a Benjamini-Hochberg 1% FDR threshold. We repeated this analysis for the identified CpG sites in [GEO: GSE48325] [20] (85 samples), selecting CpG sites that passed a Benjamini-Hochberg 1% FDR threshold. Using these criteria, we found 322 age-associated CpG sites. These sites were used to calculate entropy (Fig. 1c) for [GEO: GSE61258] [4].

Entropy was calculated according to the formula described in [1]:

$$ E n t r o p y=\frac{1}{N\times log\left(\frac{1}{2}\right)}{\displaystyle {\sum}_i^N\left[ M{F}_i\times log\left( M{F}_i\right)+\left(1- M{F}_i\right)\times log\left(1- M{F}_i\right)\right]} $$

where *MF*_*i*_ is the methylation fraction of the *i* ^th^ methylation CpG site and *N* is the number of age-associated CpG sites (393 sites for mice and 322 sites for human, described above). Since the entropy approaches 0 when *MF*_*i*_ approaches 0, the entropy for methylation sites with a value of 0 were set to 0.

### Epigenetic clock data processing and data normalization

For construction of an epigenetic-aging model, we used [GEO: GSE60012] [19], [SRA344045] [21] and our own control mice, for a total of 124 mice liver/hepatocyte samples. Because RRBS is targeted towards CpG-rich regions of the genome, we included sites that were covered by ≥2 reads in 97% of mice, mapped to chromosomes 1–19 and had a standard deviation >0 and ≤20%. Mice missing over 30% of these sites were removed from further analysis. Missing data were imputed using the mean value of each site. These filtering steps resulted in 119 samples profiled across 7628 CpG sites. For studies profiling a single time point [42] and the long-lived mice, in order to maximize the overlap with the 7628 CpG sites selected above, we considered any site with ≥1 reads (bedtools intersectbed). Missing data were imputed by the mean methylation value for that site.

All data were then normalized using ComBat (nonparametric mode) from the SVA package in R [22, 23]. Ages (in days) were transformed to log_2_ scale, prior to normalization. The specific sequencing studies ([19, 21, 42], Ames and UM-HET3) were used to represent batch, and the model provided to ComBat included the covariates age, gender and treatment. After performing ComBat, we used PCA to verify that this normalization reduced the effects due to differences in sequencing technology or mouse strains (Additional file 1: Figure S2D,E). Bismark alignment reports, as well as average read depth per unique CpG called and per CpG used to construct the epigenetic-aging model, are shown in Additional file 2: Public data and Data here detailed.

### Epigenetic-aging model construction

The normalized methylation values from [19, 21, 42] and data from wild-type, untreated UM-HET3 and Ames aged to 2 and 22 months (one from each group) (Additional file 2: Datasets used summary) were used as training data for ElasticNet regression [24] using the python scikit-learn package [52]. The normalized methylation values were used as features, and the log_2_-transformed ages (in days) were used as the predicted variable. Model fitting parameters were selected using 4-fold cross validation. The final model was trained on these training data with the most optimal regularization parameters when averaging the 4-fold cross-validation results. The model sites selected by ElasticNet, along with the associated weights and intercept, are shown in Additional file 3.

We assessed whether epigenetic ages were informative by comparing the epigenetic ages for untreated, wild-type mice from our study or mice from Cannon et al. [42]. We used either a t-test or an ANOVA to compare whether epigenetic ages were significantly different between 2- versus 22-month-old mice, and whether epigenetic ages of mice with similar chronological ages were affected by differences in genetic backgrounds (Additional file 4: Wild type stats). To assess the effect of normalization in addition to selection of regularization parameters or hidden biases correlated to aging signals, the covariates of each study were shuffled, ComBat normalization was repeated, and models were learned using the same strategy described above. This process was repeated 120 times and predictions between models generated from permuted data or actual data were compared using the residual (epigenetic age minus chronological age) for wild-type mice. The model learned from actual data minimized the residual for the wild-type mice (Additional file 1: Figure S2F–J).

We used the annotations for mouse (described above) to annotate the selected sites to genomic regions, considering only intronic, intergenic, exonic, promoter and enhancer regions. When there were overlapping annotations, we prioritized enhancer and promoter regions. We calculated under-representation of over-representation of these selected sites in these regions using a Fisher’s exact test, with significance defined as *p* < 0.01. We assigned nearest genes to these sites using closestBed and displayed this along with overlapping histone/chromatin state information in Additional file 3.

### Assessing epigenetic age in long-lived mice

The epigenetic-aging model was applied to the methylation profiles of long-lived mice and the age-matched controls not used for training (Additional file 2: Datasets used summary). Reductions in age were calculated by subtracting the epigenetic ages of the untreated, wild-type mice from those of the treated mice of the same genetic background. To assess the significance, we used an ANOVA for all 22-month-old mice or only 22-month-old UM-HET3 mice. We also compared the epigenetic ages between treatments with their age-matched controls from the same genetic background using a t-test (Additional file 4: Treatment vs wild type stats).

### Principal component analysis

PCA was conducted using scikit-learn package with the 148 CpG sites used in the epigenetic clock. The first two PCs separated age and treatment (Additional file 1: Figure S3A). We assessed the significance of variables that contributed to the variance along PC1 for each genetic background using a multivariate linear regression according to the following model:

$$ Principal\kern0.24em  component\kern0.24em 1\sim age+ treatment $$

where *treatment* was modeled as a categorical variable. Results are shown in Additional file 5.

### Hierarchical clustering

Hierarchical clustering was performed using python SciPy with linkage method “average” and Euclidean distance [53]. Methylation values were transformed using standard_scale = True and visualized using seaborn [54]. Hierarchical clustering was performed either using the top 20 most variable CpG sites (determined from the long-lived mice and wild-type mice) or all sites used by the epigenetic-aging model.

## Additional files

Additional file 1: Supplementary Figures S1–S3. Supplementary figures accompanying main text and legends. (PDF 1263 kb)

Additional file 2: Descriptions of sequencing data used to construct a mouse epigenetic-aging model. Tables describing the samples used to train and test the mouse epigenetic-aging model, and detailed alignment statistics corresponding to the samples used for analyses related to Figs 2 and 3. (XLSX 93 kb)

Additional file 3: Epigenetic-aging model with weights in the units of log_2_(days) and nearest genes. The CpG sites displayed by chromosome:start:stop (0-based) with associated weights and the intercept. These units are in log_2_(age in days). The nearest genes, distance from nearest gene (bp), and/or overlapping histone/chromatin features are reported. The directionality refers to upstream or downstream relative to the reference sequence position. (XLSX 59 kb)

Additional file 4: Epigenetic age predictions of long-lived mice and wild-type controls. Tables describing the predictions of epigenetic age for long-lived mice and wild-type controls, including summary tables describing statistical tests that were performed using this underlying data (XLSX 51 kb)

Additional file 5: The effects of age and treatments on the variance of PC1. The results of the multivariate linear regression for the independent variables age and treatment against PC1 of the 148 CpG markers used for UM-HET3 and Ames mice. (XLSX 9 kb)

Additional file 6: Weights of long-lived and wild-type control mice used in this study. Description of the weights (grams) of various control and long-lived mice according to their age. The minimum–maximum weight is described, along with the average for each age/treatment condition. (DOCX 54 kb)

## Abbreviations

CpG: Cytosine-phosphate-guanine
FDR: Fals﻿e Discovery Rate
GEO: Gene expression omnibus
OR: Odds ratio
PC: Principal component
PCA: Principal component analysis
RRBS: Reduced representation bisulfite sequencing
SRA: Sequence reads archive
TSS: Transcription start site
UTR: Untranslated region
WGBS: Whole genome bisulfite sequencing
