# Supplementary material for: Epigenetic aging signatures in mice livers are slowed by dwarfism, calorie restriction and rapamycin treatment
Source: Genome Biol. 2017 Mar 28;18:57. doi: 10.1186/s13059-017-1186-2 (PMC5371228; doi:10.1186/s13059-017-1186-2)
Supplement: Supplementary file 1 — Supplementary Figures S1–S3. Supplementary figures accompanying main text and legends. (PDF 1263 kb) [file 13059_2017_1186_MOESM1_ESM.pdf]

Additional File 1: Supplementary Figures (Figure S1-S3)

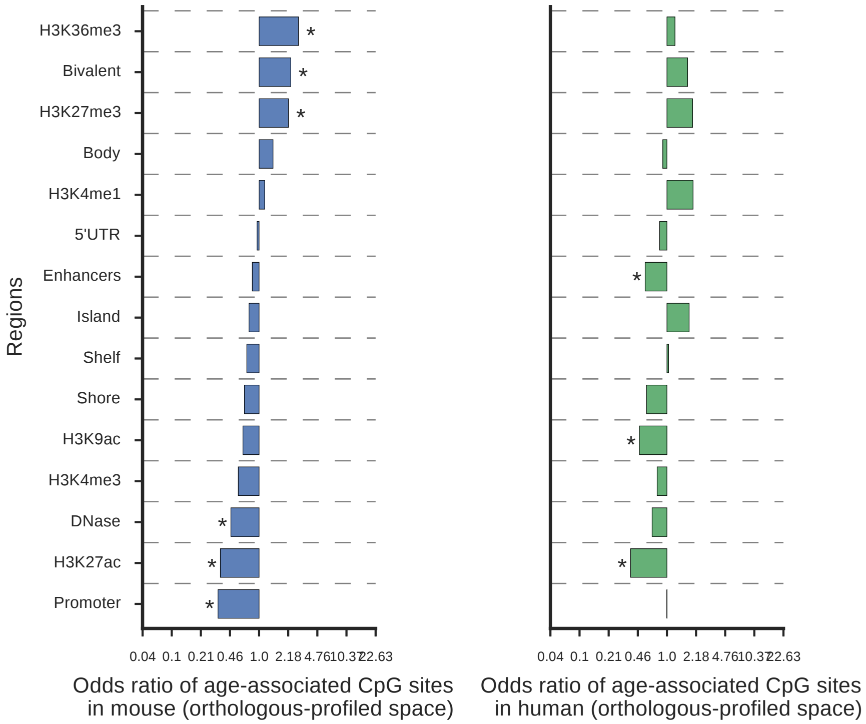
Regions
H3K36me3
Bivalent
H3K27me3
Body
H3K4me1
5'UTR
Enhancers
Island
Shelf
Shore
H3K9ac
H3K4me3
DNase
H3K27ac
Promoter
0.04 0.1 0.21 0.46 1.0 2.18 4.76 10.37 22.63
Odds ratio of age-associated CpG sites in mouse (orthologous-profiled space)
Odds ratio of age-associated CpG sites in human (orthologous-profiled space)

Figure S1: Patterns of genomic regions affected by age-associated CpG sites

Odds ratios showing enrichment (OR > 1) or depletion (OR < 1) of age-associated CpG sites in the orthologous-profiled space for different genomic feature annotations (regions) in mice (left) or humans (right). * indicates $p < 0.002$.

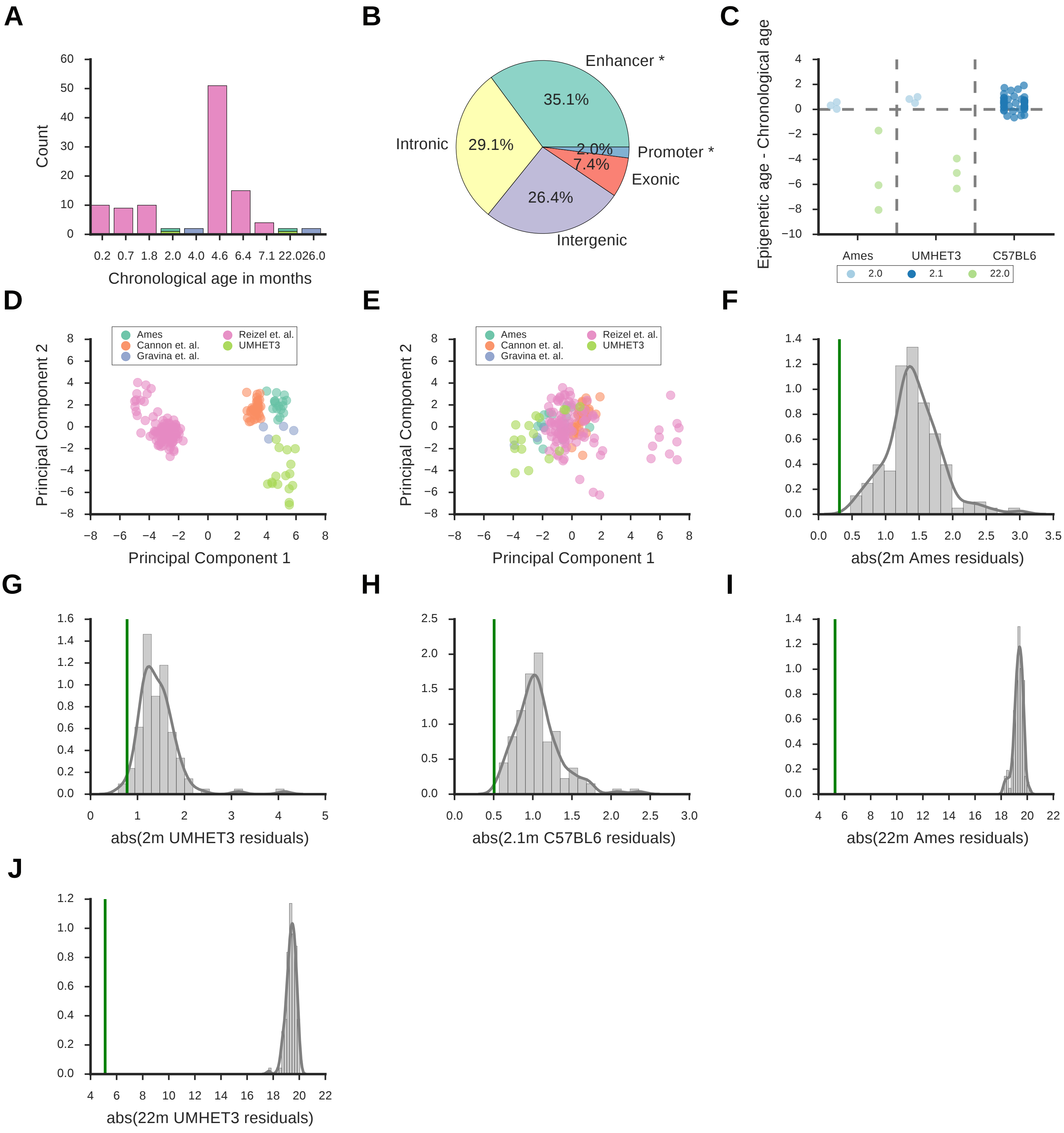

A
Count
Chronological age in months
0.2 0.7 1.8 2.0 4.0 4.6 6.4 7.1 22.0 26.0
B
Enhancer *
35.1%
Intronic
29.1%
2.0%
Promoter *
7.4%
Exonic
26.4%
Intergenic
C
Epigenetic age - Chronological age
Ames
UMHET3
C57BL6
2.0
2.1
22.0
D
Ames
Cannon et. al.
Gravina et. al.
Reizel et. al.
UMHET3
Principal Component 2
Principal Component 1
E
Ames
Cannon et. al.
Gravina et. al.
Reizel et. al.
UMHET3
Principal Component 2
Principal Component 1
F
abs(2m Ames residuals)
G
abs(2m UMHET3 residuals)
H
abs(2.1m C57BL6 residuals)
I
abs(22m Ames residuals)
J
abs(22m UMHET3 residuals)

Figure S2: Details of data processing, model sites and model quality control. A) Total counts of the number of mice according to their chronological ages in months used to train an ElasticNet regression (107 total). Colors correspond to sequencing studies, as shown in (**D, E**). **B)** The genomic features associated with the 148 CpG sites used in the model. There was an under-representation of these CpG sites in promoters and over-representation in enhancer regions. * indicates $p < 0.01$ by Fisher's exact test. **C)** Residual errors (epigenetic age - chronological age) of wild type, untreated mice of our own study and mice of Cannon *et al.* There was no detected difference in epigenetic ages across various mouse strains. Colors correspond to chronological age in months. **D)** Principal component analysis (PCA) of the 7,628 CpG sites in 173 mouse samples considered across all studies before ComBat normalization. Colors indicate the specific sequencing studies. **E)** PCA of the 7,628 CpG sites in 173 mouse samples considered across all studies after ComBat normalization, colors correspond to sequencing studies and are identical to those in (**A, D**). **F-J)** Show the results of randomizing the assignment of covariates within each study before normalizing with ComBat. After permutation and normalization, models are trained to predict age. For each permutation, models learned are then tested on the untreated, wild type mice from our study and the mice of Cannon *et al*. Residual errors are calculated for each permutation and averaged according to age and mouse genetic background. The gray bars show the distribution of average residual errors for the randomizations, where the green line indicates the residual error from the model learned on actual data. **F)** The absolute average residual error of 2-month Ames wild type mice **G)** of 2-month untreated, wild type UM-HET3 mice, **H)** of 2.1-month C57BL/6 mice, **I)** of 22-month Ames wild type mice, **J)** of 22-

C57BL/6 mice, **I)** of 22-month Ames wild type mice, **J)** of 22-month untreated, wild type UM-HET3 mice. m: months

A

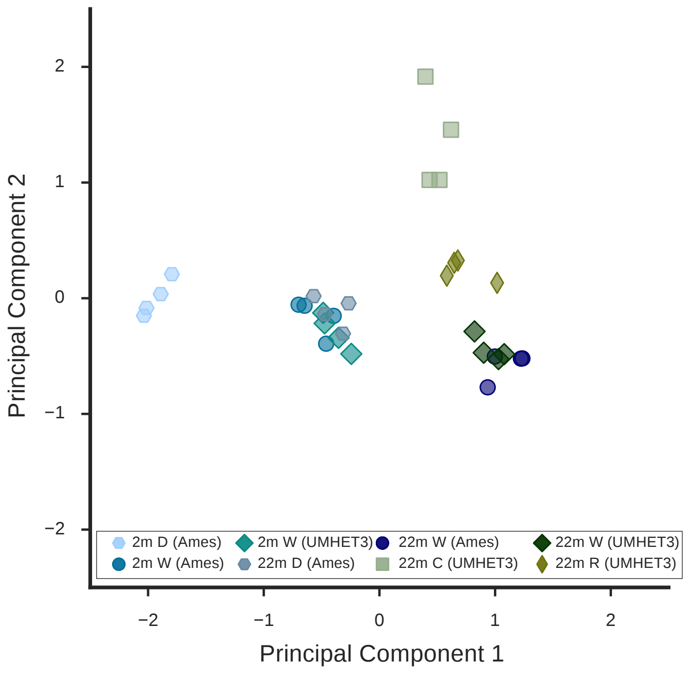

Principal Component 2
Principal Component 1
2m D (Ames)
2m W (Ames)
2m W (UMHET3)
22m D (Ames)
22m W (Ames)
22m C (UMHET3)
22m W (UMHET3)
22m R (UMHET3)

B

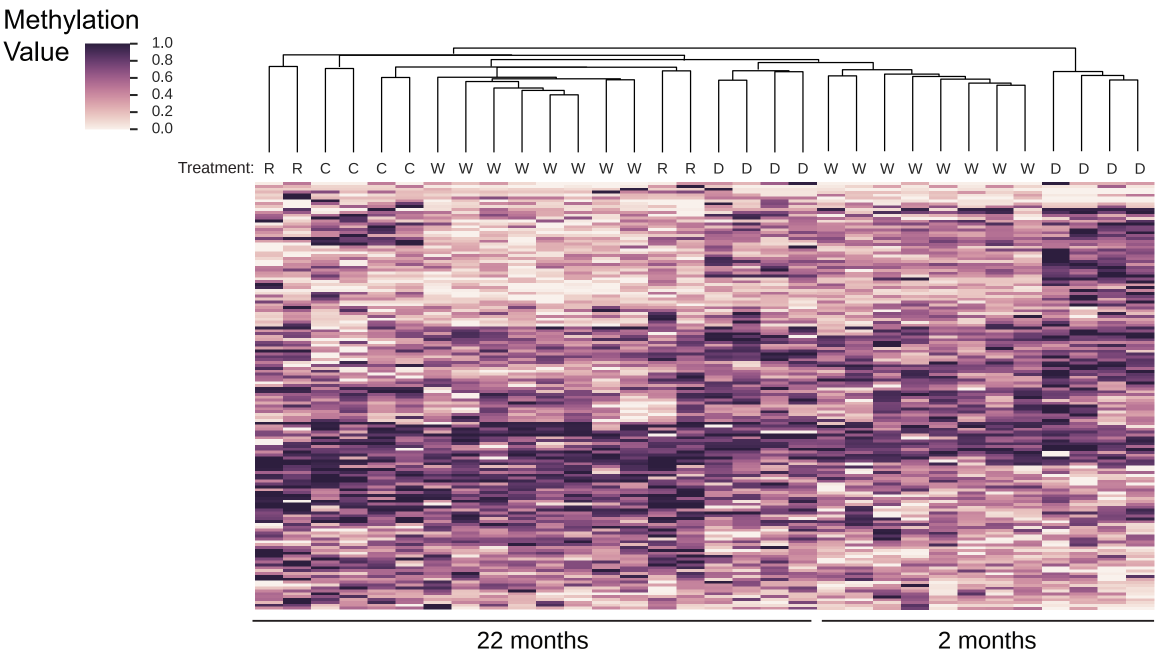

Methylation Value
1.0
0.8
0.6
0.4
0.2
0.0
Treatment: R R C C C C W W W W W W W W R R D D D D W W W W W W W W D D D D
22 months
2 months

Figure S3: Unsupervised analysis of CpG sites used in epigenetic age predictor

A) PCA of all 148 CpG sites used in ElasticNet markers is shown for the first two principal components. The colors and shapes refer to the age, treatment and mouse strain indicated in the legend. **B)** Hierarchical clustering is performed identically as performed in **Figure 3E**, except for all 148 CpG markers. Treatments are labeled beneath the dendogram and the ages are indicated at the bottom of the heatmap. m: months, R: rapamycin treated, C: calorie restricted, W: untreated, wild type Ames or UM-HET3
