# Supplementary material for: Epigenetic aging signatures in mice livers are slowed by dwarfism, calorie restriction and rapamycin treatment
Source: Genome Biol. 2017 Mar 28;18:57. doi: 10.1186/s13059-017-1186-2 (PMC5371228; doi:10.1186/s13059-017-1186-2)
Supplement: Supplementary file 6 — Weights of long-lived and wild-type control mice used in this study. Description of the weights (grams) of various control and long-lived mice according to their age. The minimum–maximum weight is described, along with the average for each age/treatment condition. (DOCX 54 kb) [file 13059_2017_1186_MOESM6_ESM.docx]

**Additional File 6:** Weights of long-lived and wild type control mice used in this study

| Ames mice |  |  |  |
| --- | --- | --- | --- |
| **Genotype** | **Age** | **Min - max weight (grams)** | **Average** |
| dwarf | 2 | 6.1-7.9 | 7.2 |
| wildtype | 2 | 20.7-25.8 | 23 |
| dwarf | 22 | 15.3-28.2 | 23.3 |
| wildtype | 22 | 22.1-39.3 | 33.6 |

| UM-HET3 mice | |  |  |
| --- | --- | --- | --- |
| **Treatment** | **Age** | **Min - max weight (grams)** | **Average** |
| untreated | 2 | 21.9-24.1 | 23.2 |
| untreated | 22 | 29.8-47.1 | 35.6 |
| calorie restricted | 22 | 19.7-23 | 21.2 |
| rapamycin treated | 22 | 29.8-47.1 | 35.6 |
